# Comparison of the 1-Year Visual and Anatomical Outcomes between Subthreshold Red (670 nm) and Yellow (577 nm) Micro-Pulse Laser Treatment for Diabetic Macular Edema

**DOI:** 10.3390/ph14111100

**Published:** 2021-10-28

**Authors:** Wataru Kikushima, Taiyo Shijo, Yukiko Furuhata, Yoichi Sakurada, Kenji Kashiwagi

**Affiliations:** Department of Ophthalmology, University of Yamanashi, Chuo 409-3898, Yamanashi, Japan; tshijo@yamanashi.ac.jp (T.S.); fyukiko@yamanashi.ac.jp (Y.F.); sakurada@yamanashi.ac.jp (Y.S.); kenjik@yamanashi.ac.jp (K.K.)

**Keywords:** diabetic macular edema, subthreshold red micropulse laser treatment, subthreshold yellow micropulse laser treatment, diabetic retinopathy

## Abstract

We investigated the efficacy and safety of red (670 nm) subthreshold micropulse laser (SMPL) treatment for diabetic macular edema (DME) and compared the 1-year treatment outcomes of red and yellow (577 nm) SMPL for DME. A medical chart review was performed in 43 consecutive eyes of 35 patients who underwent red or yellow SMPL treatment for DME and were followed up for 12 months. There were 26 and 17 eyes in the yellow and red SMPL groups, respectively. The mean best-corrected visual acuity (BCVA) was maintained throughout the follow-up period of 12 months in the yellow and red SMPL groups (*p* = 0.39, *p* = 0.70, respectively). The central retinal thickness (CRT) measured by spectral-domain optical coherence tomography (SD-OCT) was significantly decreased at 12 months from baseline in the yellow and red SMPL groups (*p* = 0.047, *p* = 0.03, respectively). Although the amount of CRT reduction in the red SMPL group was significantly greater than that in the yellow SMPL group at 8 months from baseline (*p* = 0.02), the significance disappeared at the final follow-up period (*p* = 0.44). The red SMPL maintained the BCVA in patients with center-involving DME. The mean CRT in the red SMPL group significantly decreased, and the amount of CRT reduction was equivalent to that in the yellow SMPL group.

## 1. Introduction

Diabetic macular edema (DME) is a complicated pathology that can appear even in the early stage of diabetic retinopathy and can cause a mild to moderate reduction in the visual acuity of the patients [1]. Several treatment options have been reported in past studies to handle DME, including partial photocoagulation [2], sub-Tenon’s/intravitreral triamcinolone acetonide injection [3], intravitreal anti-vascular endothelial growth factor (VEGF) agent injection [4,5], vitrectomy [6,7], intravitreal dexamethasone implant [8,9], and combinations of these treatment protocols [10]. Subthreshold micropulse laser treatment (SMPL) was first reported by Friberg and Karatza as a non-invasive laser treatment directly targeting the macular area with a wavelength of 810 nm (infrared SMPL) for DME [11]. Many studies have reported favorable results of infrared SMPL for treating DME, both functionally and anatomically [12,13,14,15,16,17,18,19,20]. Yellow SMPL with a wavelength of 577 nm is another option of SMPL and has been compared with infrared SMPL in several studies [21,22,23,24,25,26]. Few studies reported the comparison of the effects of SMPL and other treatment options for DME. Wu et al. reported that the combination of intravitreal anti-VEGF therapy and conventional laser photocoagulation therapy was more effective than infrared SMPL monotherapy in improving the vision of patients with DME [27]. On another front, Akkaya et al. reported that yellow SMPL treatment was superior to intravitreal anti-VEGF therapy in DME patients with relatively better BCVA for increasing visual acuity and decreasing central retinal thickness (CRT) for 12 months [28]. However, there is no consensus regarding the optimal wavelength of SMPL for DME. This study aimed to investigate the efficacy and safety of a novel treatment protocol of SMPL with a wavelength of 670 nm (red SMPL) for DME and compare the visual and anatomical outcomes of red and yellow SMPL for DME.

## 2. Results

### 2.1. Changes in logMAR BCVA

**Figure 1:**
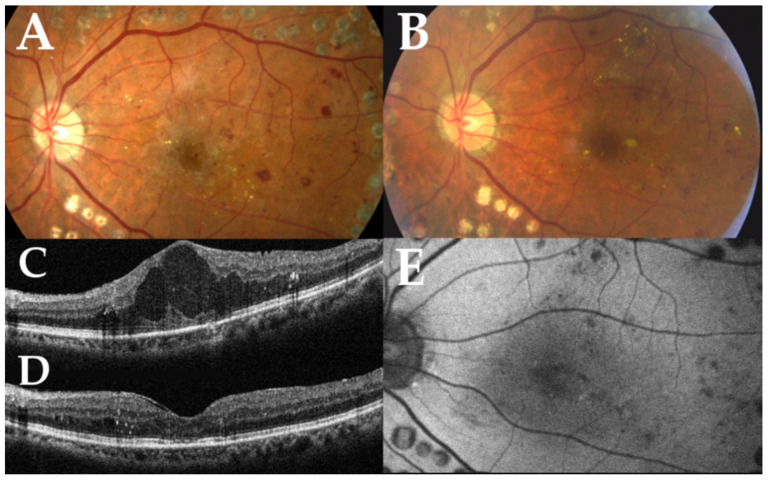
A representative case of a 57-year-old male with diabetic macular edema (DME) treated by red subthreshold micropulse laser (SMPL). (**A**) Color fundus photography of the left eye at baseline reveals center-involving DME. His left visual acuity was 0.7 on the decimal scale. (**B**) Color fundus photography of the left eye at 12 months from baseline reveals complete resolution of DME. No visible laser spot is detectable. His left vision improved to 1.0 on the decimal scale. (**C**) A spectral-domain optical coherence tomography (SD-OCT) image of the left macula at baseline demonstrates the cystoid macular edema (CME) with a disrupted ellipsoid zone. Central retinal thickness (CRT) was 673 µm. (**D**) An SD-OCT image of the left macula at 12 months from baseline demonstrates complete resolution of CME and improved integrity of the ellipsoid zone. CRT drastically decreased to 237 µm. (**E**) Fundus autofluorescence of the left macula at 12 months from baseline shows no visible laser spot. Dark spots around the fovea represent retinal hemorrhages shown in the color fundus photograph (**B**).

**Figure 2:**
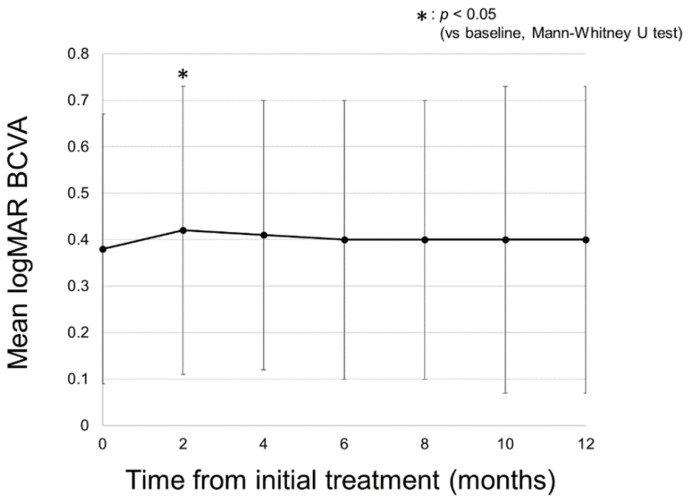
Changes in the mean logarithm of the minimal angle resolution (logMAR) best-corrected visual acuity (BCVA) in all patients. The mean BCVA in all patients was 0.38 ± 0.29 at baseline and was 0.42 ± 0.31, 0.41 ± 0.29, 0.40 ± 0.30, 0.40 ± 0.33, and 0.40 ± 0.33 at 2, 4, 6, 8, 10, and 12 months, respectively. The mean logMAR BCVA was consistent at 12 months from baseline (*p* = 0.55).

**Figure 3:**
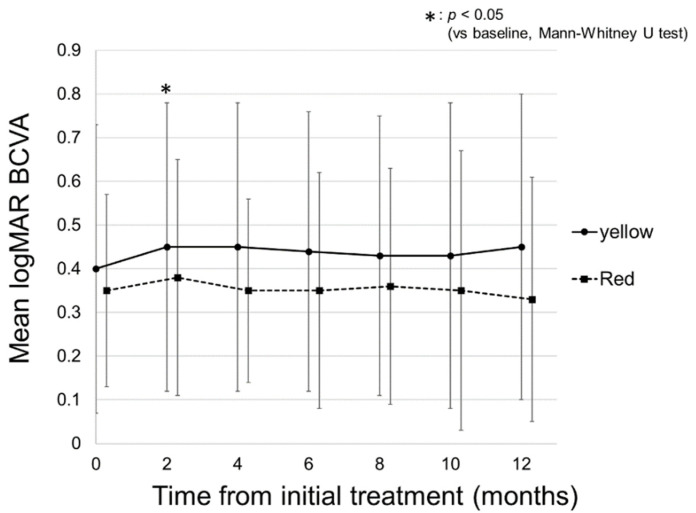
Changes in the mean logarithm of the minimal angle resolution (logMAR) best-corrected visual acuity (BCVA) in the yellow and red subthreshold micropulse laser (SMPL) groups. In the yellow SMPL group, the mean BCVA was 0.40 ± 0.33 at baseline and was 0.45 ± 0.33, 0.45 ± 0.33, 0.44 ± 0.32, 0.43 ± 0.32, 0.43 ± 0.35, and 0.45 ± 0.35 at 2, 4, 6, 8, 10, and 12 months, respectively. There was no significant difference between the mean BCVA at baseline and each follow-up period other than 2 months (*p* = 0.015, 0.069, 0.34, 0.39, 0.53, and 0.39, respectively). In the red SMPL group, the mean BCVA was 0.35 ± 0.22 at baseline and was 0.38 ± 0.27, 0.35 ± 0.21, 0.35 ± 0.27, 0.36 ± 0.27, 0.35 ± 0.32, and 0.33 ± 0.28 at 2, 4, 6, 8, 10, and 12 months, respectively. There was no significant difference between the mean BCVA at baseline and each follow-up period (*p* = 0.42, 1.00, 0.93, 0.78, 0.91, and 0.70, respectively).

A total of 43 eyes of 35 patients were included in this study. Table 1 shows the baseline characteristics of the patients and a comparison of those in the yellow and red SMPL groups. There were 26 eyes in the yellow SMPL group and 17 eyes in the red SMPL group. There was no significant difference in the baseline characteristics between the two groups. Figure 1 shows a representative case of DME treated with red SMPL. Figure 2 shows the change in the mean logarithm of the minimal angle resolution (logMAR) best-corrected visual acuity (BCVA) in all patients. The mean BCVA in all patients was 0.38 ± 0.29 at baseline, maintained throughout the follow-up period, and was 0.40 ± 0.33 at 12 months (*p* = 0.55). Figure 3 shows the change in the mean logMAR BCVA between the two groups. In the yellow SMPL group, the mean BCVA was 0.40 ± 0.33 at baseline, which was maintained at 12 months from baseline, resulting in a mean BCVA of 0.45 ± 0.35 (*p* = 0.39). In the red SMPL group, the mean BCVA was 0.35 ± 0.22 at baseline and was maintained throughout the follow-up period, resulting in 0.33 ± 0.28 at 12 months from baseline (*p* = 0.70).

We performed a multivariate linear regression analysis to investigate the visual prognostic factors associated with BCVA at 12 months. As a result, only baseline logMAR BCVA was associated with the final BCVA at 12 months (shown in Table 2).

### 2.2. Changes in CRT on SD-OCT

**Figure 4:**
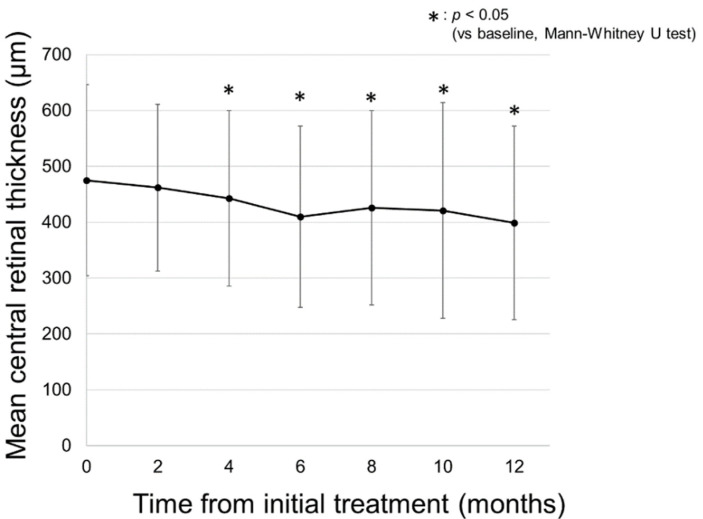
Changes in the mean central retinal thickness (CRT) measured with spectral-domain optical coherence tomography in all patients. The mean CRT in all patients was 475 ± 171 µm at baseline and was 462 ± 149, 443 ± 157, 410 ± 162, 426 ± 174, 421 ± 193, and 399 ± 173 µm at 2, 4, 6, 8, 10, and 12 months, respectively. The mean CRT in all patients significantly decreased at 4 months from baseline, and the significant reduction was maintained throughout the 12 months of the follow-up period (*p* = 0.41, 0.04, 0.003, 0.02, 0.03, and 0.003, respectively).

Figure 4 demonstrates the change in the mean central retinal thickness (CRT) on spectral-domain optical coherence tomography (SD-OCT) in all patients. The mean CRT on SD-OCT in all patients was 475 ± 171 µm at baseline and significantly decreased to 443 ± 157 µm at 4 months from baseline (*p* = 0.04). The significant reduction in CRT remained consistent until 12 months from baseline (399 ± 173 µm, *p* = 0.003).

**Figure 5:**
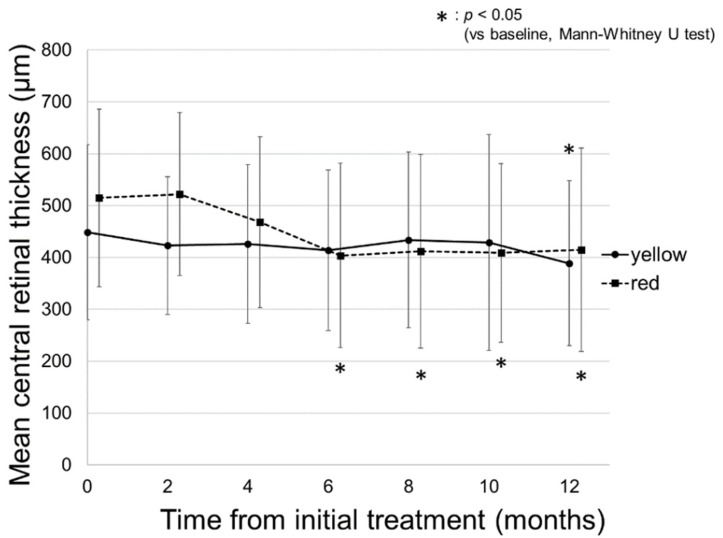
Changes of in the mean central retinal thickness (CRT) in the yellow and red subthreshold micropulse laser (SMPL) groups. The mean CRT in the yellow SMPL group was 449 ± 169 µm at baseline and was 423 ± 133, 426 ± 153, 414 ± 155, 434 ± 169, 429 ± 208, and 389 ± 159 µm at 2, 4, 6, 8, 10, and 12 months from baseline. In the yellow SMPL group, the mean CRT remained consistent from baseline to 10 months, and significantly reduced at 12 months (*p* = 0.30, 0.23, 0.17, 0.54, 0.55, and 0.047, respectively). The mean CRT in the red SMPL group was 515 ± 171 µm at baseline and was 522 ± 157, 468 ± 165, 404 ± 178, 412 ± 187, 409 ± 172, and 415 ± 196 µm at 2, 4, 6, 8, 10, and 12 months from baseline. A significant reduction in CRT in the red SMPL group was observed at 6 months and remained consistent for 12 months from baseline (*p* = 0.63, 0.11, 0.004, 0.01, 0.005, and 0.03, respectively).

Figure 5 shows the change in the mean CRT in the yellow and red SMPL groups. The mean CRT in the yellow SMPL group was 449 ± 169 µm at baseline and remained consistent from baseline until 10 months (429 ± 208 µm, *p* = 0.55). At the final visit after 12 months, the mean CRT in the yellow SMPL group significantly reduced to 389 ± 159 µm (*p* = 0.047).

The mean CRT in the red SMPL group showed a clinical course similar to that of the yellow SMPL group. The mean CRT was 515 ± 171 µm at baseline and significantly reduced to 404 ± 178 µm at 6 months (*p* = 0.004). Thereafter, the significant reduction in CRT in the red SMPL group remained consistent for 12 months (415 ± 196 µm, *p* = 0.03, shown in Figure 5).

Among the patients, four eyes (15.4%) in the yellow SMPL group and six eyes (35.3%) in the red SMPL group showed complete resolution of DME at 12 months. There was no significant difference between the proportion of the patients with complete resolution of DME at 12 months in the yellow and red SMPL groups (*p* = 0.13, chi-square test).

**Figure 6:**
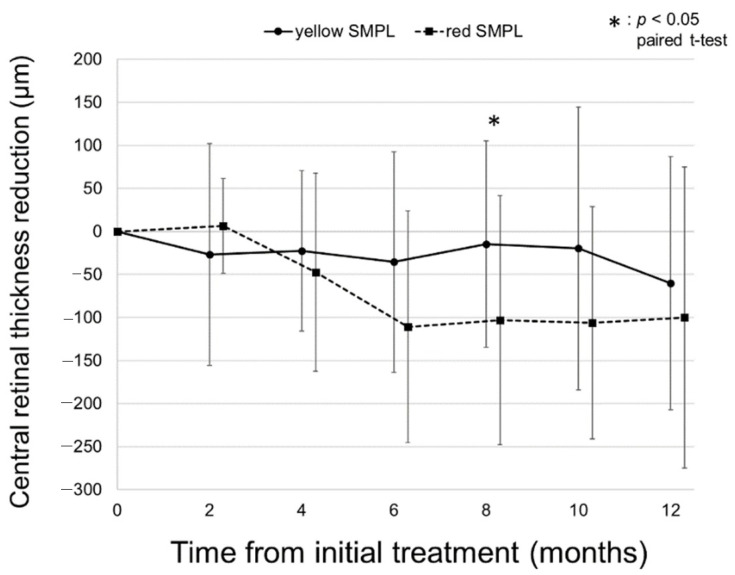
Comparison of the mean central retinal thickness (CRT) reduction in the two groups. There was no significant difference in the mean CRT reduction between the yellow and red subthreshold micropulse laser (SMPL) groups throughout the 12 months of the follow-up period except at 8 months from baseline, when the mean CRT reduction in the red SMPL group was greater than that in the yellow SMPL group (*p* = 0.58, 0.65, 0.09, 0.02, 0.10, and 0.44 at 2, 4, 6, 8, 10, and 12 months from baseline).

To investigate the difference in the treatment effect of CRT reduction for DME between the yellow and red SMPL groups, we compared the mean CRT reduction in the two groups. As a result, there was no significant difference in the mean CRT reduction between the yellow and red SMPL groups throughout the 12 months of the follow-up period, except at 8 months from baseline, when the mean CRT reduction in the red SMPL group was greater than that in the yellow SMPL group (*p* = 0.02, shown in Figure 6).

To investigate the factors associated with CRT at 12 months, we conducted a multivariate linear regression analysis. As a result, only baseline CRT was associated with final CRT at 12 months (shown in Table 3).

### 2.3. Number of Total SMPL Treatments and Evaluation of the Safety

Table 4 presents the values of the parameters for the yellow and the red SMPL treatment during the 12 months of the follow-up period. During the 12 months of the follow-up period, the mean number of total SMPL treatments in all patients was 5.1 ± 1.1. There was no significant difference in the mean number of total SMPL treatments between the yellow and red SMPL groups (*p* = 0.48, shown in Table 4). The fundus autofluorescence (FAF) images showed no visible spots in the treated area in either group (shown in Figure 1).

## 3. Discussion

In this study, we compared the 1-year treatment outcomes of yellow and red SMPL for center-involving DME. As a result, SMPL with 577- and 670-nm wavelengths significantly reduced macular edema and maintained the BCVA in patients with DME. To the best of our knowledge, this is the first study to report the treatment outcomes of red (670 nm) SMPL for DME.

Several studies have reported on the visual outcomes of SMPL for DME. Vujosevic et al. compared the two treatment modalities of SMPL (yellow and infrared) for mild DME (<400 µm) [21]. They reported that BCVA was consistent at 6 months from baseline in both treatment groups. Recently, Al-Barki et al. compared the short-pulse subthreshold and infrared micropulse macular laser for DME [29] and reported that the infrared micropulse macular laser yielded a significant improvement in the BCVA in patients with DME. In contrast, the short-pulse subthreshold laser maintained the BCVA up to 6 months from baseline. Additionally, in other literature, a significant improvement in the BCVA was reported after SMPL [14,17,23]. In this study, mean BCVA was maintained throughout the 12 months of the follow-up period in both the yellow and red SMPL groups. One possible reason for the difference in the visual outcomes between this study and these previous studies is the difference of the proportion of treatment-naïve patients. The fact that 70% of the patients in this study had a history of treatment for DME might have affected the visual outcome.

In the literature, a number of the favorable anatomical outcomes of SMPL have been reported [14,19,25]. In the previous study described above, Vujosevic et al. revealed the anatomical outcomes after yellow and infrared SMPL. Although the mean CRT significantly decreased at 6 months in the yellow micropulse laser (MPL) group, there was no significant difference in CRT change between the yellow and infrared MPL groups [21]. In the prospective study, Al-Barki et al. reported that the mean CRT significantly decreased by 32 µm in the whole cohort; however, in the individual study arms, a significant reduction in the CRT was observed in the short-pulse subthreshold EndPoint 50% laser group only. Similar to those of previous studies, the anatomical outcomes of red SMPL in this study were favorable. The amount of CRT reduction was significantly greater in the red SMPL group at 8 months from baseline and tended to be greater (but not significant) than in the yellow SMPL group thereafter. This might be due to the difference in baseline CRT between the two groups.

In this study, despite a significant reduction of CRT, no significant improvement in BCVA was observed after SMPL. We consider that there are some possible reasons for this discrepancy. One possible reason is the high proportion of patients with past treatment history for DME. Valera-Cornejo et al. recently reported the short-term treatment outcomes of yellow MPL in eyes with either treatment-naïve or refractory DME [30]. They revealed no significant change in the BCVA at 3 months in both groups and a significant reduction in the CMT at 3 months was observed in the treatment-naïve group only. In accordance with the previous study, the visual and anatomical outcomes between eyes with treatment-naïve and past-treated DME in our study cohort were equivalent (data not presented). However, the cumulative damage of photoreceptors caused by persistent DME might have restricted the visual recovery. Another possible reason is the difference in the range of baseline CRT between the patients in previous studies and those in this study. In our study cohort, patients with mild to severe DME (range of CRT: 207–796 µm) were included in the analysis, and despite the relatively thicker CRT, a significant reduction in CRT was achieved in both SMPL groups. However, the wide range of baseline CRT in this study might have confused the visual outcome.

In the present study, the mean number of total SMPL treatment was around 5 in both groups during the follow-up period. This means that most of the patients received additional SMPL at every follow-up visit, whereas many previous reports showed similar treatment outcomes of SMPL with fewer number of total treatments. In the previous study, Vujosevic et al. reported that most of the patients received yellow or infrared SMPL twice during 6 months [21]. Furthermore, in most of the published studies, only one or two SMPLs were performed [17,19,20,25]. We consider that the possible reason for the relatively large number of additional treatments in this study was the small number of laser spots per one SMPL (99.7 ± 9.8, Table 4). The number of laser spots per one SMPL varies depending on the studies, ranging approximately from 150–500. The smaller number of laser spots in this study might have encompassed the insufficient treatment efficacy.

We consider that the advantages of red SMPL over yellow SMPL are better penetration and less scattering, enabling better delivery to the target tissue. In addition, the 670-nm laser is not absorbed by xanthophyll or hemoglobin, resulting in minimal adverse effects on the neurosensory retina [31,32,33]. However, the multivariate linear regression analyses revealed that the difference in the treatment modality (yellow or red SMPL) was not associated with the final BCVA or CRT at 12 months. In this study, the safety of red SMPL was confirmed by the FAF images of the macular area treated by several repeated SMPLs. Therefore, we consider that this novel treatment modality for SMPL is a promising option for center-involving DME, especially with a good initial BCVA.

Our study has some limitations. The first limitation was the retrospective nature of the analysis. The second limitation was the relatively small sample size. The third limitation was the proportion of treatment-naïve patients, as aforementioned. Further prospective studies with a larger study population are necessary to confirm the efficacy and safety of red SMPL.

## 4. Materials and Methods

### 4.1. Participants

A retrospective medical chart review was conducted for the eyes of patients who received either red or yellow SMPL for DME and were followed up for at least 12 months. This study was approved by the Institutional Review Board of the University of Yamanashi (protocol code: 2089 and the date of approval: 26 July 2019) and was conducted following the tenets of the Declaration of Helsinki. Written informed consent was obtained from all participants during the study period.

The inclusion criteria were as follows: (1) eyes with treatment-naïve DME or DME not treated within the last 4 months to allow for a sufficient wash-out period; (2) center-involving DME with a CRT >200 µm measured by vertical or horizontal images of SD-OCT; and (3) BCVA >0.05 on a decimal scale. The exclusion criteria were as follows: (1) eyes with any treatment history for DME within the last 4 months; (2) eyes with a previous history of vitrectomy; (3) eyes with macular edema caused by other retinal pathologies (branch retinal vein occlusion, central retinal vein occlusion, age-related macular degeneration, or macular telangiectasia); (4) other ocular abnormalities that affect the central visual field, including glaucoma, optic neuritis, macular hole, epiretinal membrane, or severe cataract; and (5) systemic disorders that affect CRT, including renal failure, heart failure, or hemodialysis.

At baseline, all of the participants underwent comprehensive ophthalmic examinations including measurement of BCVA using Landolt chart, intraocular pressure, dilated fundus examination with slit-lamp biomicroscopy, color fundus photography, fluorescein angiography (FA), and SD-OCT examination (OCT-HS100, Cannon Lifecare Solutions, Kanagawa, Japan).

### 4.2. SMPL Treatment Protocol

Patients received SMPL with a wavelength of either 577 (yellow SMPL) or 670 nm (red SMPL) once and followed up bimonthly. All SMPLs were performed by three retinal specialists (WK, TS, and YF) using a TruScan Laser (LIGHTMED, San Clemente, CA, USA). The Mainster Focal/Grid contact lens (Ocular Instruments, Bellevue, WA, USA) was applied to the cornea of the patients after pupillary dilation and topical anesthesia. In both protocols, the subthreshold laser power was determined by titrating the burn to a barely visible spot with a continuous laser and then the titrated value was set with SMPL mode. Yellow SMPL treatment was performed with the following settings: a wavelength of 577 nm, 200 µm spot size on the slit lamp (magnified to 210 µm on the retina), 10% duty cycle of 0.2 s, 120–350 mW power (lower than the titrated laser power making barely visible spot), 80–120 spots to cover the macular area and varying according to the extension of DME. Red SMPL treatment was performed with the following settings: a wavelength of 670 nm, 200 µm spot size on the slit lamp (magnified to 210 µm on the retina), 10% duty cycle of 0.2 s, 120–350 mW power (lower than the titrated laser power making the barely visible spot), 80–120 spots to cover the macular area and varying according to the extension of DME. In both protocols, the laser was applied repeatedly and continuously to cover the entire area of DME (spacing at 0).

Yellow or red SMPL treatment was chosen depending on the time period. Yellow SMPL was performed as the initial treatment (yellow SMPL group) from September 2017 to July 2019, and red SMPL was performed as the initial treatment (red SMPL group) from August 2019 to February 2020.

After receiving the initial SMPL treatment, all patients were followed up bimonthly. Retreatment of SMPL with the same treatment protocol as that of initial SMPL was performed if the CRT was ≥400 µm, the reduction of the CRT was <50% from baseline, or there was no improvement in the BCVA. In addition, to evaluate the safety of the SMPL treatment, all patients underwent FAF examination at 6 and 12 months from baseline.

### 4.3. Statistical Analysis

The primary outcome measures included BVCA improvement and CRT reduction at 12 months after baseline. BCVA was measured on a decimal scale and converted into a logMAR. All statistical analyses were conducted using the Statflex 7 software (Artec Co., Ltd., Osaka, Japan). The differences in categorical and continuous variables between the yellow and red SMPL groups were analyzed using the chi-square test, Mann–Whitney U test, or Kruskal–Wallis test. In addition, the paired t-test was used to compare the significance of the differences between the values before and after treatment. A *p*-value of <0.05 was considered as statistically significant.

## 5. Conclusions

The red SMPL with a wavelength of 670 nm maintained the BCVA of patients with center-involving DME. The mean CRT in the red SMPL group significantly decreased, and the amount of CRT reduction was equivalent to that in the yellow SMPL group.

## Figures and Tables

**Table 1:** Baseline characteristics of the patients and a comparison of those in the yellow and red SMPL groups.

	All Patients (*n* = 43)	Yellow SMPL (*n* = 26)	Red SMPL (*n* = 17)	*p* Value (Yellow vs. Red)
Age	66.0 ± 10.1	65.3 ± 11.4	67.1 ± 7.9	0.97
Gender Male (%)	29(67.4%)	18(69.2%)	11(64.7%)	0.76
Duration of DM (months)	121 ± 100	134 ± 106	100 ± 90	0.28
Hb A1c (%)	7.0 ± 1.2	7.0 ± 1.4	6.9 ± 0.8	0.82
Mean BCVA	0.38 ± 0.29	0.40 ± 0.33	0.35 ± 0.22	0.96
Mean CRT	475 ± 171	449 ± 169	515 ± 171	0.19
Treatment-naïve eye (%)	14(32.6%)	8(30.8%)	6(35.3%)	0.76

BCVA: best-corrected visual acuity, CRT: central retinal thickness, DM: diabetes mellitus, Hb A1c: hemoglobin A1c, SMPL: subthreshold micropulse laser.

**Table 2:** Multivariate linear regression analysis of factors associated with mean BCVA at 12 months.

Variables	β-Coefficient	*p*-Value
Age	−1.3 × 10^−4^	0.98
Male gender	−0.04	0.64
Baseline logMAR BCVA	0.77	1.0 × 10^−5^
Baseline CRT	−8.6 × 10^−5^	0.73
HbA1c	0.067	0.055
Duration of DM	1.4 × 10^−5^	0.97
Past treatment history for DME	0.02	0.78
SMPL (yellow: 0, red: 1)	−0.07	0.42

BCVA: best-corrected visual acuity, CRT: central retinal thickness, DM: diabetes mellitus, DME: diabetic macular edema, logMAR: logarithm of the minimal angle resolution, SMPL: subthreshold micropulse laser.

**Table 3:** Multivariate linear regression analysis of factors associated with mean CRT at 12 months.

Variables	β-Coefficient	*p*-Value
Age	−0.39	0.88
Male gender	−20.5	0.70
Baseline logMAR BCVA	154.1	0.10
Baseline CRT	0.47	3.4 × 10^−3^
HbA1c	−1.4	0.94
Duration of DM	−0.13	0.62
Past treatment history for DME	51.9	0.33
SMPL (yellow: 0, red: 1)	1.0	0.98

BCVA: best-corrected visual acuity, CRT: central retinal thickness, DM: diabetes mellitus, DME: diabetic macular edema, logMAR: logarithm of the minimal angle resolution, SMPL: subthreshold micropulse laser.

**Table 4:** Values of the parameters for the yellow and red subthreshold micropulse laser treatment during the 12 months of the follow-up period.

	All Patients (*n* = 43)	Yellow SMPL (*n* = 26)	Red SMPL (*n* = 17)	*p* Value (Yellow vs. Red)
Mean laser power (mW)	269 ± 57	228 ± 19	327 ± 41	<1.0 × 10^−6^
Mean number of laser spots	99.7 ± 9.8	102.1 ± 10.0	96.3 ± 8.6	1.0 × 10^−5^
Mean (Median) number of total SMPL treatment	5.1 ± 1.1 (6.0)	5.1 ± 1.1 (5.0)	5.2 ± 1.1 (6.0)	0.48

SMPL: subthreshold micropulse laser.

## Data Availability

The data presented in this study are available on request from the corresponding author. The data are not publicly available due to their containing information that could compromise the privacy of research participants.
